# How do older adults with multimorbidity navigate healthcare?: a qualitative study in Singapore

**DOI:** 10.1186/s12875-023-02195-2

**Published:** 2023-11-14

**Authors:** Poay Sian Sabrina Lee, Evelyn Ai Ling Chew, Hui Li Koh, Stephanie Xin En Quak, Yew Yoong Ding, Mythily Subramaniam, Janhavi Ajit Vaingankar, Eng Sing Lee

**Affiliations:** 1 Clinical Research Unit, National Healthcare Group Polyclinics, 3 Fusionopolis Link #06-13, Singapore, 138543 Singapore; 2 Department of Geriatric Medicine, Tan Tock Seng Hospital, Singapore, Singapore; 3 Research Division, Institute of Mental Health, Singapore, Singapore; 4 Lee Kong Chian School of Medicine, Nanyang Technological University, Singapore, Singapore

**Keywords:** Multimorbidity, Navigation, Older adults, Qualitative

## Abstract

**Background:**

Patients living with multimorbidity may require frequent visits to multiple healthcare institutions and to follow diverse medical regimens and advice. Older adults with multimorbidity could face additional challenges because of declining cognitive capability, frailty, increased complexity of diseases, as well as limited social and economic resources. Research on how this population navigates the healthcare system in Singapore also remains unknown. This study investigates the challenges older adults with multimorbidity face in navigating healthcare in Singapore.

**Methods:**

Twenty older adults with multimorbidity from a public primary care setting were purposively sampled. Interviews conducted inquired into their experiences of navigating the healthcare system with multiple conditions. Inductive thematic analysis was performed by independent coders who resolved differences through discussion.

**Results:**

Older adults with multimorbidity form a population with specific characteristics and challenges. Their ability to navigate the healthcare system well was influenced by these themes including patient-related factors (autonomy and physical mobility, literacy and technological literacy, social support network), healthcare system-related factors (communication and personal rapport, fragmented system, healthcare staff as advocate) and strategies for navigation (fitting in, asking for help, negotiating to achieve goals, managing the logistics of multimorbidity).

**Discussion:**

Older adults with multimorbidity should not be treated as a homogenous group but can be stratified according to those with less serious or disruptive conditions (less burden of illness and burden of treatment) and those with more severe conditions (more burden of illness and burden of treatment). Among the latter, some became navigational experts while others struggled to obtain the resources needed. The variations of navigational experiences of the healthcare system show the need for further study of the differential needs of older adults with multimorbidity. To be truly patient-centred, healthcare providers should consider factors such as the existence of family support networks, literacy, technological literacy and the age-related challenges older adults face as they interact with the healthcare system, as well as finding ways to improve healthcare systems through personal rapport and strategies for reducing unnecessary burden of treatment for patients with multimorbidity.

**Supplementary Information:**

The online version contains supplementary material available at 10.1186/s12875-023-02195-2.

## Background

Minimally Disruptive Medicine (MDM) [1] proposes that when the demands imposed by the healthcare system exceed patients’ capacity to cope, they decompensate. Patients living with multimorbidity, i.e., multiple chronic diseases, often shoulder a heavy burden of illness (BoI) and burden of treatment (BoT) as they may require frequent visits to multiple venues and follow diverse medical regimens and advice. While studies have sought to identify, measure and reduce the BoT of such patients [2–8], much remains to be done [9], and research on how this population navigates the healthcare system in non-Western parts of the world remains scarce.

Healthcare navigation is one aspect of the experience of patients with multimorbidity that can pose considerable challenges for patients [10–16]. Navigation is defined as “the process(es) by which patients and/or their health caregivers move into and through the multiple parts of the healthcare enterprise in order to gain access to and use its services in a manner that maximises the likelihood of gaining the positive health outcomes available through those services.” [17] Known navigational challenges for patients with multimorbidity include managing multiple appointments; long waiting times in healthcare premises; poor communication between different doctors or clinics, and transportation issues, especially for the less mobile patients [18, 19].

The healthcare system in Singapore is a combination of public and private practitioners. Primary public healthcare is provided by more than 20 polyclinics (over 300 doctors) which are healthcare centres that provide primary care such as medical treatment, preventive healthcare, and health education, at a subsidised cost which is supported by the Ministry of Health, Singapore. Primary private healthcare is run by General Practitioners (GPs) with 1,700 private clinics (over 2,500 doctors). Primary healthcare professionals help to coordinate patients’ care with other providers and help patients to navigate the healthcare system. Patients are at liberty to choose among a variety of healthcare providers depending on their needs, preferences, accessibility, resources and other personal reasons. Despite the preponderance of private GPs, majority of patients with chronic conditions prefer to visit the polyclinics.

To date, there is limited published research specifically on healthcare navigation and older adults with multimorbidity or other patient groups in Singapore. Foo and colleagues [20] examined the facilitators and barriers of managing patients with multiple chronic conditions in the community in Singapore. However, to date, there is limited published research specifically on healthcare navigation and older adults with multimorbidity or other patient groups in Singapore. Another study by Koh et al., [21] explored the hassles that patients with chronic diseases in the primary care setting faced. The authors found that patients with multimorbidity reported higher degree of hassles than those with one chronic disease. From a previous published study in Singapore [22], it was demonstrated that prevalence of multimorbidity increases with age. In addition, the reason to explore healthcare navigation in older adults with multimorbidity is because they may face different challenges in navigating the healthcare system. Their views would shine some light on how healthcare system can be improved in terms of healthcare system processes and designs. Therefore, this study seeks to uncover the healthcare navigational experiences and challenges specific to older adults with multimorbidity in Singapore.

## Theoretical framework

**Fig. 1:**
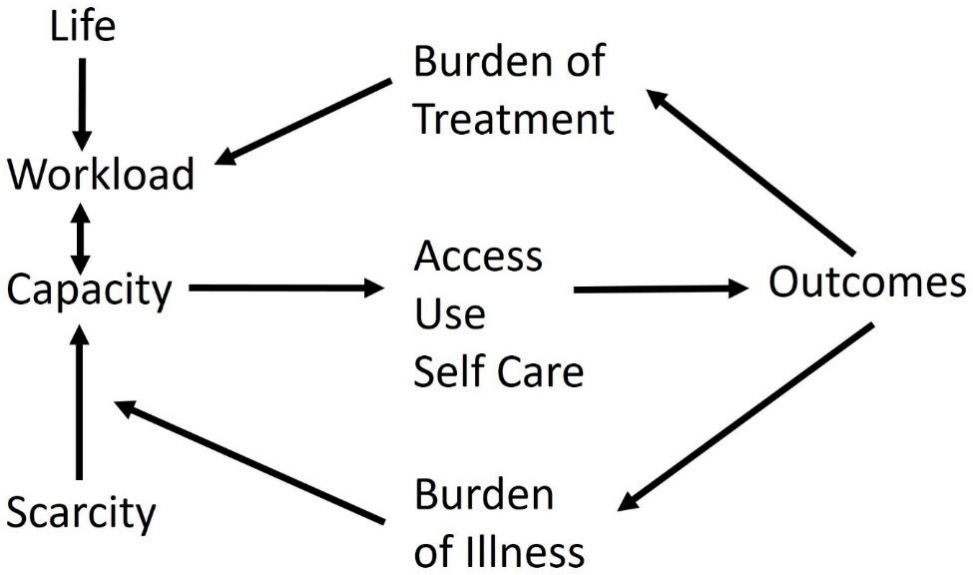
Cumulative complexity model (Adopted from Boehmer et al., 2016 and Shippee et al., 2012)

MDM is a patient-centred approach to care which focuses on achieving patients’ goals for life and health while imposing minimum burden of treatment on their lives. The aim of MDM is to reduce the workload and increase capacity for patients with multimorbidity [9]. The MDM concept is based on the cumulative complexity model (CCM) where it acknowledges that in patients with multimorbidity, the workload of healthcare is often moderated by the capacity to handle the work [9]. (Fig. 1)

The present study utilises the concepts of MDM and cumulative complexity to shed light upon the challenges faced by older adults with multimorbidity in navigating the healthcare system.

Based on two theories, the interview guide (Additional File 1) was designed to elicit older adults with multimorbidity’s experiences in three areas of navigation: Physical navigation (getting to and from appointments, moving around the healthcare venue), logistical navigation (appointment and medication tracking and management), and information access (ability to gain access to information relevant to their healthcare needs). The study team members have designed the interview guide iteratively after several rounds of discussions with further inputs from family physicians within the study team. For the questions related to “different conditions”, since each participant has a different list of chronic conditions that he/she suffered from, they were written down in the interview guide. For each interview, the interviewer will specifically refer to the chronic conditions, unique to individual participants.

## Methods

This qualitative description study [23–25], underpinned by a realist, naturalistic epistemological approach, explored the navigational experiences of older adults with multimorbidity within the healthcare system in Singapore, which is largely based on a fee-for-service healthcare financing model. The research design and methods of this study are described in detail in our protocol [26].

The inclusion criteria were older adults with three or more chronic conditions between the ages of 60–99 years old. There was no pre-defined list of chronic conditions. Patients who were able to verbalise three or more chronic conditions followed up with healthcare providers were eligible for the study. The definition of chronicity of a disease as lasting at least six months, having a documented pattern of recurrence or deterioration, and having an impact on an individual’s quality of life [27] was adopted. They would also require medical follow-ups with National Healthcare Group Polyclinics (primary care) and at least one specialist outpatient clinic at any restructured hospitals for the past one year. The participants needed to be conversant in English, Mandarin, Malay or Tamil. The study excluded patients who were unable to provide informed consent due to cognitive impairment, inability to communicate through speech or writing, or do not wish to be audio-recorded.

These patients were purposively sampled for diversity by ethnicity, educational status and age, and were referred to the study by attending clinic staff. Study team members had no prior personal relationship with any of the interviewees. Interviewees were informed of the purpose of the study and given time to decide freely whether to participate.

Trained study team members (a male medical doctor/researcher and four female research staff) conducted semi-structured individual interviews lasting approximately one hour, either in a quiet room within the healthcare institution, or at participants’ homes. All interviewers were experienced researchers studying multimorbidity. Interviews were audio-recorded and transcribed verbatim. Unstructured field notes were taken by researchers to aid in the analysis, and NVivo 12 (QSR International, Australia) was used to organise the data. Data analysis was run concurrently, and recruitment ceased when thematic saturation was judged to have been reached [26, 28].

Inductive thematic analysis was carried out by researchers in the study team trained in qualitative methodology. Preliminary initial codes were derived from three coders independently coding the first two transcripts. These initial codes were applied to a third transcript, and this process was used to refine the coding frame. Coding for the remaining transcripts was divided among the coding team of six, who worked in pairs, using the revised coding frame. To strengthen intercoder agreement, all transcripts were separately coded by at least two team members, who met periodically to check their coding with each other. The entire coding team also met regularly to discuss and propose new codes that emerged during analysis, and to modify existing codes when needed. The process was iterative, moving between coding and revising the codes. Any major revisions to the coding frame were agreed upon by all members of the coding team. Eventually, four members of the coding team revisited all the coded transcripts to ensure consistency in application of the codes.

Initial analysis yielded a total of 108 initial codes (Additional File 2). Discussion among the study team led to streamlining the codes into 23 broader codes (Additional File 3). After further discussion, codes not directly related to the research question were removed, and others combined. Finally, the ten remaining codes were sorted into three broad areas relating to patient navigation—patient-related factors, healthcare system-related factors, and strategies for navigation.

## Results

Twenty-one older adults with multimorbidity were interviewed between February 2019 and April 2021 (Table 1). One participant was excluded as she was discovered to have a mild cognitive impairment and was unable to articulate her conditions. A more detailed patient profile is presented in Additional File 4.

**Table 1:** Participants’ Characteristics

Participants’ characteristics (n = 20)		
Gender	Male	13
	Female	7
Ethnicity	Chinese	12
	Malay	4
	Indian	4
Age	60–69	6
	70–79	10
	> 80	4
Education Level	No Formal Education	4
	Primary School	5
	Secondary School	9
	Pre-U / College/ University	2

### Overview of results

Although we did not explicitly seek to categorise our interviewees, we discovered during analysis that the older adults with multimorbidity interviewed fell along a spectrum: Some reported minor or negligible challenges in navigation, while others struggled with various aspects of managing their conditions and obtaining needed resources.

Older adults with multimorbidity who reported that they had “no problems” and “no issues” navigating (e.g., P002, P003, P006) had fewer and well-controlled conditions. The most common conditions reported were hypertension, diabetes and hyperlipidemia. When these conditions were mild or borderline and stable, the impact on older adults with multimorbidity’s lives remained relatively minor. They had fewer medications and appointments, interacted with the healthcare system less frequently—sometimes only once in six months per condition—and reported fewer navigational difficulties.

Older adults with multimorbidity with more severe, more numerous or poorly controlled conditions (e.g., P007, P010, P012) typically had more frequent interactions with the healthcare system. The most extreme case, P007, needed dialysis and diabetic wound care, and would visit a healthcare venue almost daily. This group generally experienced more challenges in navigating the healthcare system in order to attain their health goals. Nevertheless, there was not a direct association between severity or number of conditions and difficulties navigating. Some older adults with multimorbidity were able to manage relatively well because they had adequate resources to support them, while others struggled to overcome various navigational challenges.

In short, the older adults with multimorbidity could be loosely categorised as follows: (1) those with well-controlled conditions who reported minor to no navigational difficulties, (2) those with clinically severe conditions and hence considerable burden of illness, who had developed navigational skills and had sufficient resources to support them, and (3) those distressed by a heavy burden of illness and burden of treatment (similar to the previous category), but with difficulties in achieving their navigational goals (e.g., managing appointments or accessing relevant information). The differences between the second and third groups can be accounted for by the patient-related factors we discuss subsequently.

**Table 2:** Summary of Themes

**Patient-Related Factors**	1. Autonomy and physical mobility 2. Literacy and technological literacy 3. Informal social support network
**Healthcare System-Related Factors**	4. Communication and personal rapport 5. Fragmented system 6. Healthcare staff as advocate to facilitate navigation
**Strategies for Navigation**	7. Fitting in 8. Asking for help 9. Negotiating to achieve own goals 10. Managing logistics (appointment tracking and medication management)

As shown in Table 2, older adults with multimorbidity’s navigational abilities were influenced by patient-related factors such as: Level of autonomy and physical mobility, literacy and technological literacy, and the presence or absence of a strong social support network. Certain aspects of the healthcare system were also mentioned by older adults with multimorbidity as factors that either helped or hindered their navigation. These included: Healthcare staff acting as advocates, communication and personal rapport, and fragmentation in the healthcare system. Faced with challenges to navigation, older adults with multimorbidity reacted by either fitting in (adapting to the situation), asking for help, or negotiating to achieve their desired goals.

### A. patient-related factors

Older adults with multimorbidity who managed their conditions well had conditions which were less disruptive of their lives, such as hypertension, hyperlipidaemia and heart conditions which had stabilised. Consequently, their BoI and BoT were relatively light. They were also highly literate, mobile, and had strong social support networks. These older adults with multimorbidity frequently took a proactive approach to their illnesses: monitoring and recording their blood pressure or glucose daily, seeking information online or attending public seminars to learn about their conditions.

At the opposite extreme were older adults with multimorbidity who found navigating the healthcare system more problematic. They tended to be technologically averse, illiterate or poorly educated, and lacking in social support from friends and family. They also tended to take a passive attitude to knowing about and managing their medications and conditions. The remaining older adults with multimorbidity fell between the two poles, with some showing strong desires to manage their health but sometimes facing communication difficulties or other obstacles.

#### 1. Autonomy and physical mobility

Chronic pain and difficulties with physical mobility were navigational challenges that afflicted about one third of the older adults with multimorbidity. Three older adults with multimorbidity (P005, P007, P013) related how leg pain interfered with their lives and with getting to medical appointments. P013 usually requested that his appointments be in the morning, as his legs would fail him in the afternoon. P007 and P016 sometimes had to change or cancel their appointments when they felt too unwell to leave the house. For mobility challenged older adults with multimorbidity, a wheelchair enabled independent movement, but created new challenges. Not all taxis and buses would take wheelchair-bound passengers, and the physical trajectory to the clinic was in some cases too bumpy for wheelchair use.

Some older adults with multimorbidity found age-related forgetfulness a navigational challenge. P003 failed to show up for an appointment because he had forgotten, while P013 arrived for a Monday morning appointment to find the clinic closed, because it was in fact a Sunday. A few reported forgetting to take their medications, or got confused by changes to their accustomed medical regimen:

*“That doctor asked me to stop: ‘You take this for one week and then you stop.’ I carried on. I came back to him, he said, “You’re supposed to have stopped!” I forgot!” (P006)*.

Although navigational problems caused by forgetfulness could have been solved by relying on a caregiver to keep track of their appointments, older adults with multimorbidity expressed a strong desire to maintain their independence and were reluctant to rely on others. They preferred not to “trouble” their children or even the full-time domestic helpers their adult children had hired to look after them.

#### 2. Literacy and Technological literacy

A key navigational challenge raised by some older adults with multimorbidity was the extent to which the Singapore healthcare system relies upon patients or caregivers being able to read and to use technology. Most older adults with multimorbidity felt adept at navigating the healthcare system by asking questions in person but balked at navigating the Internet or automated phone systems. Online appointment booking systems presented obstacles for those with little formal education or those unfamiliar with the Internet. Older adults with multimorbidity who were illiterate (P002, P004, P005, P009) could not read text message appointment reminders sent to them by healthcare institutions, nor access health-related information online. Moreover, not all older adults with multimorbidity had ready access to the Internet. As P006 protested, “you cannot assume everyone has Internet.”

Technological anxiety was also a hindrance to navigation. P005 rang the phone hotline when she forgot the date of her next appointment but felt helpless to navigate the automated voice system and hung up. She ended up missing the appointment: “Everything is using that machine to talk. I don’t know what to ask.” P013 was likewise confounded by the automated phone answering systems:

*One thing is, that we don’t know technology: “Please press 1. For Mandarin speaking, press 2.” You have to press, then it will say a whole lot of words, inform you about some promotion, [which I] also don’t understand. And then, if you press the wrong thing, then no more [the call ends]. [sigh]*

Others expressed frustration at being unable to get through to a human being on the phone hotline. Rather than attempting to navigate the online appointment booking system or the automated phone system, some older adults with multimorbidity preferred to walk into the clinic despite longer waiting time without prior-arranged appointments.

### 3. Informal Social Support Network

Family support played a large role in determining how well a patient successfully navigated the system to achieve optimal health outcomes. Some older adults with multimorbidity relied completely on caregivers to navigate the healthcare system. P007, P015, and P016 admitted that they could not survive without their caregivers. P007, with severe diabetes, heart disease, and end-stage renal failure, depended almost entirely on his wife, who fed him, accompanied him to appointments, manoeuvred his wheelchair on public transport, and advocated for his rights when he had been mistreated. P016’s niece accompanied him to all his appointments and communicated with the healthcare institutions. P015 even opined that if his wife were to go on strike as his caregiver, he would die:

I’ve also said this before, if [she] dies first, I will follow, follow [her] there. Because [about] all the medications I know nothing. Appointments, I don’t know, I don’t want to remember them.

### 3a. Adult children as “Bridge” to communicate with Healthcare System

Children of older adults with multimorbidity often helped them navigate the technological environment, gain access to relevant information, and generally acted as a bridge between the older adults with multimorbidity and the healthcare system—translating messages or information from English into their spoken language or dialect, changing appointments over the phone or online, and so on.

Older adults with multimorbidity with limited social support faced greater navigational challenges. For instance, P009, estranged from his immediate family, had no stable dwelling and shuttled between living with a cousin and with a friend. He was unemployed and had few friends. Unable to read and with no one to accompany him, he remained unaware of some resources available to him, such as a free shuttle bus service that could facilitate his moving around the healthcare venue. In contrast, most other older adults with multimorbidity who were illiterate had children who informed them about available resources and helped communicate with the healthcare institution.

### 3b. “Not wanting to Bother”

Paradoxically, some older adults with multimorbidity avoided accessing social resources available to them. At least four older adults with multimorbidity disclosed concern about being a burden to their loved ones. Consequently, some refrained from informing their children about their appointments or financial needs, so as not to inconvenience their children or oblige them to provide financial help: “Sometimes [they] spend half a day to accompany me, [it’s as if they] don’t need to work already!” (P002) “They have their own lives, right? So, I don’t want to trouble them and ask them for money, to see doctor or anything … they have their own families, we don’t go and trouble them.” (P015).

### B. Healthcare System-related factors

Besides factors that were directly related to the patient, older adults with multimorbidity identified factors relating to the healthcare system which affected their healthcare navigation.

#### 4. Communication, “Presence” and Personal Rapport

Aspects of good communication that older adults with multimorbidity felt helped them in their navigation included the ability to explain health-related information in a way older adults with multimorbidity could understand, personal rapport, and sufficient time and attention given to patients.

Older adults with multimorbidity appreciated healthcare professionals who took time to explain and educate them on their conditions. This facilitated older adults with multimorbidity’s navigation by making available relevant healthcare information and increasing their health literacy.

*We get the [test] results, the graph and all these things we don’t know how to read. Whether good or not, we don’t know. So the doctor will check and then they explain to you. … sometimes you don’t know what the medicine, why you must take this medicine … so they will explain to you. (P003)*

Some doctors resorted to drawings or non-verbal gestures to help explain older adults with multimorbidity’s health situation to them. P013 was grateful that his doctor explained, using gestures, how the tumour in his intestine was obstructing his gut and why he urgently needed an operation. Another understood his atherosclerosis and the need for dietary adjustments after the doctor explained it with a drawing. When older adults with multimorbidity understood their conditions, they felt better able to take responsibility for their own health and act accordingly.

Good patient-doctor rapport, based upon clear and supportive communication, positively influenced patient navigation/ access of healthcare information. Some older adults with multimorbidity had very good personal rapport with the healthcare team and knew their doctors by name. When older adults with multimorbidity felt healthcare professionals knew them personally and gave them time to ask questions, they felt happier and ‘encouraged’, leading to greater trust in the healthcare system and greater confidence in their ability to navigate it.

In contrast, some older adults with multimorbidity complained that doctors focused too much on the on-screen medical records and *“never even look at you”* (P003, P005), and *“just look at the monitor”* (P005, P002). The perceived condescension and lack of interest led some older adults with multimorbidity to keep their questions to themselves:

*“I don’t think the doctor want to discuss with you—you may not be able to understand the medical concepts… So normally they don’t discuss.” (P006)*.

Likewise, P005, who was not well educated, opined:

Even if you want to tell him anything there’s no chance, because if he doesn’t ask you, you cannot reply, right?

Negative encounters arising from perceived lack of time or attention from healthcare professionals resulted in a lack of trust. Patients who disagreed with their doctors, or whose needs were not met, would turn to a different doctor or institution. P005, who suffered from chronic leg pain as well as diabetes, hypertension, Parkinson’s and other illnesses, felt the doctor she first saw was dismissive:

*“They don’t care about my leg”*.

Internally disagreeing with the doctor’s referral to a physiotherapist, she consulted a private clinic where she had regular, expensive injections for her leg. The injections did not definitively resolve her problem, although they provided some symptomatic relief, and she felt her needs had been met.

In short, perceptions of healthcare professionals and healthcare institutions and past experiences with the healthcare system influenced the way older adults with multimorbidity navigated and accessed health care.

#### 5. Fragmented system resulting in increased burden of treatment or non-attendance

Other systemic issues that hindered patient navigation arose from the fragmented nature of the healthcare system. P009 was scheduled two appointments less than an hour apart at different healthcare institutions. Unable to attend both, he opted to miss his lung appointment in favour of seeing the skin doctor for his chronic rash, which was a more pressing problem for him.

Older adults with multimorbidity with multiple frequent appointments struggled to juggle several appointments at different venues. Along with dialysis thrice a week at the dialysis centre and diabetic wound dressings at the primary care clinic twice a week, P007 juggled specialist appointments for his heart, kidney, and eye conditions. To reduce travel expenditure, he would try to combine multiple appointments on one day in the same hospital, which left him exhausted.

A strategy employed by some older adults with multimorbidity, of combining multiple appointments into a single trip to reduce hassle travel expenses, led to a different problem—that of long gaps between appointments, where they had nowhere to “be”. While some older adults with multimorbidity did not mind this, those in a delicate state of health, such as P007, mentioned earlier, who had end-stage kidney disease, diabetes and was wheelchair-bound, found the long waits draining and frustrating. Even older adults with multimorbidity with relatively lower BoI and BoT found their multiple visits burdensome and time-consuming.

#### 6. Healthcare Staff as advocates within Healthcare System

Older adults with multimorbidity’s navigation of the healthcare system was facilitated by staff who acted as advocates for them, helping them overcome barriers to their healthcare navigation goals, such as obtaining an appointment with a specialist or an earlier appointment.

When P010 who suffered from anxiety and depression told his psychiatrist that he did not want to take his medicine because it was too expensive, the psychiatrist found a workaround by discharging him to primary care where he could receive subsidised medication. Likewise, P007 reported that his year-long, futile attempts to obtain an appointment with a specialist were quickly resolved when a primary care doctor came to his aid with a referral note:

I don’t know what he wrote, [but] immediately, they fixed an appointment—on the same day.

As part of the healthcare system, clinicians and other institutional staff were able to help older adults with multimorbidity access institutional resources more easily. A medical social worker helped solve P007’s travel difficulties, too. Getting up the bus was problematic for the wheelchair bound P007, but he had no money to take taxis to all his many appointments. The social worker started a process to obtain taxi vouchers for him and, at the same time, found an ambulance to shuttle him between his home and the healthcare venue. Other older adults with multimorbidity such as P015 and P017 likewise recalled occasions where staff recommended them to financial assistance programmes or other helpful resources within the healthcare system when needed.

### C. Strategies for Navigation

In the face of barriers to navigation, older adults with multimorbidity developed strategies such as ‘fitting in’ to the system, asking for help, or negotiating to achieve their own goals. Most older adults with multimorbidity also had their own medication and appointment management strategies to cope with the demands of the patient workload imposed by multimorbidity.

#### 7. “Fitting In”

Several older adults with multimorbidity adapted to navigational challenges by treating the situation as immutable, and adapting themselves to the existing system or situation, without challenging or seeking to change it. Thus, P013 avoided questioning items on the medical bill that he did not understand. P005 kept her medical doubts to herself instead of clarifying them, because she felt the doctor had no time for her. P002 would take a bus journey the long way around to get to the clinic to reduce walking because of weakness and pain in his legs.

P012 exemplifies this attitude when he describes patiently waiting in the clinic:

*“I just follow. Even [if it’s] one hour also I wait. I don’t go and knock or ask the nurse”.*

Nonetheless, this “good patient” behaviour [29] was combined with the savviness of knowing how to “fit in” to his advantage: By arriving earlier than his appointment, he knew that he could have his appointment brought forward if an earlier slot became available.

Some ‘fitting in’ strategies ended up compounding hassles for the patient. For instance, P007’s strategy of combining several appointments in a day to reduce costs meant leaving home before daybreak to avoid peak hour traffic, arriving hours before his scheduled appointment and remaining in the same hospital until his last appointment. Consequently, some of his visits to a healthcare institution lasted more than 12 h.

#### 8. Asking for help

A contrasting navigational strategy was to “ask for help” regarding medicines, directions or processes, as a form of self-advocacy. For several older adults with multimorbidity, asking for help was their first recourse. P009, who could not read, routinely approached staff in his town council for help in interpreting the text messages sent to his mobile phone by the healthcare institution.

After repeated interactions with the healthcare system, some older adults with multimorbidity had become adept at knowing where to get resources. P004 knew that in an asthma attack, he could get fast-track access to a nebuliser by asking the nurse for help, instead of waiting in line (“fitting in”). P007 knew he could ask to have an appointment brought forward if an earlier slot became vacant.

Asking for help navigating the physical environment seemed to be the most self-evident manner of navigating for P010:

You got a mouth, you ask. Definitely you will get it. If you keep quiet, you can go nowhere.

P002 who was also illiterate, likewise opined:

[People nowadays] can read, they understand everything. People like us, since we are not educated, we need to ask in order to get directions.

#### 9. Negotiating to achieve own goals

Older adults with multimorbidity who became expert navigators through repeated interactions with the healthcare system had learned to negotiate with the system to achieve goals important to them. When P007 was told he needed to have his leg amputated, he was highly reluctant and negotiated with the doctors to try to save the leg:

*“After emphasising, talk to him for half an hour … I said you see [when] my soul leaves this world I don’t want to lose any parts of my body”.*

The doctor agreed to hold off the amputation and perform an angioplasty to try to save the leg.

Another patient (P013) persuaded the doctor to put him on medical leave as he felt it would be dangerous for him to be working as a driver owing to his symptoms on that day. A third patient felt his medication regimen was “too ridiculous” and negotiated with his doctor to reduce the intake to twice a day.

*“In one day, I eat about… 20 plus, 30 medicines. Morning, night. I don’t take any in the afternoon. Initially I was supposed to take in the afternoon, but I don’t take. Because it’s too ridiculous already, to have to take it even in the afternoon. I told the doctor. Doctor said ok, [how about] you take it at night”. (P015)*

#### 10. Managing the Logistics of Multimorbidity—a “Full-Time Job”

Besides strategies such as ‘fitting in’, ‘asking for help’ and ‘negotiating to achieve one’s own goals’, another aspect of older adults with multimorbidity’s experiences of navigating healthcare related to the background work of keeping tabs on appointment times and venues, how much medicine to take home and how to administer it.

Depending on older adults with multimorbidity’s health conditions and the resulting medication regimens and frequency of visits, managing appointments and medicines required an array of logistical supports, leading P012 to describe his illness as a “full time job”. Bulletin boards, wall calendars, phone calendars and even filing systems were frequently listed as part of older adults with multimorbidity’s management of appointments, bills and pills. Many also relied on appointment text message reminders sent by the healthcare institution. Others enlisted the help of family members such as spouses or children in tracking their appointments.

A recurring concern for some older adults with multimorbidity, which could add to their BoT, was the fear of running out of medication before their next visit. Others described the workload of organising their many medications: P007 had to take ten pills a day, P008 and P013 had 16 medications which had to be taken at various times during the day; P010 had 15.

*“You have to take all these medicine and know how many times a day… Even now I pack 3 types of medicine, 3 types of drugs. One type 3 times a day, another type 2 times a day, another one is only once a night. So must know which colour.” (P006)*.

Logistically, keeping up with multiple medication schedules posed a challenge for some older adults with multimorbidity. Some older adults with multimorbidity skipped doses or self-adjusted their medication regimen, because they found the timings prescribed too much of a hassle. P005 gave up on following her recommended medicine regimen, citing incompatibility with her lifestyle:

It’s very hard, very hard to [get the timing right]. Then … halfway through my meal [I realised] I didn’t take that medicine. Now you just ate, you cannot take. The doctor … said, “You have to wake up at 9 o’ clock to take the medicine. After taking it, you go back to sleep. [laughs] I don’t do that. I’m already awake, why would I go back to sleep?

P013 explained it was important to organise the medication into sets to be taken every day:

If you don’t organise it—if say you’re free and you don’t put it in order, at night when you want to take your medicines it will be troublesome.

For others such as P016 and P011, this task landed on their primary caregivers, who would pack each day’s medicine into a small packet or bottle to prevent patients from taking the wrong medication or dosage.

## Discussion

This study sought to discover how older adults with multimorbidity navigate the healthcare system with the aim of identifying the limitations experienced by them and strategies deployed to manage them. The BoI can reduce older adults with multimorbidity’s capacity to cope, and, by extension, the ability to navigate the healthcare system well [1]. Hence, one would expect older adults with multimorbidity with more conditions and more severe conditions to have more difficulty navigating. Indeed, older adults with multimorbidity with more severe conditions did experience more BoI and corresponding BoT, as did patients with life-disrupting conditions such as a relentless itch or chronic pain. However, not all older adults with multimorbidity with serious illnesses had difficulty navigating, as some became expert navigators as a result of their past experiences and struggles in getting around the healthcare system.

**Fig. 2:**
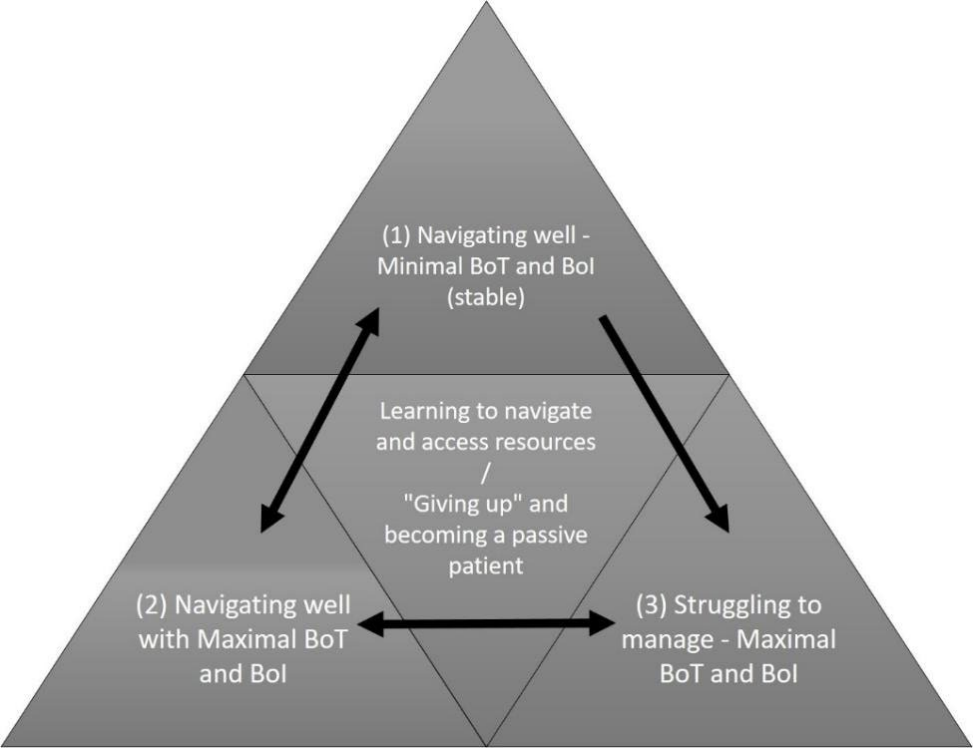
Relationship between Burden of Treatment, Burden of Illness and Older Adults with Multimorbidity’s Ability to Navigate the Healthcare System

Older adults with multimorbidity’s ability to navigate the healthcare system well was influenced by personal factors such as their physical mobility and autonomy, literacy and technological literacy, systems of social support from friends and family, as well as system-side factors. Our findings indicate that older adults with multimorbidity should not be treated as a homogeneous group but can be stratified loosely according to those with less serious or disruptive conditions (less BoI and BoT), and those with more severe conditions (more BoI and BoT). Among the latter, some became navigational experts while others struggled to obtain the resources needed. The navigational journey of an older adult with multimorbidityover time can be described in three stages, summarized in Fig. 2.

Older adults with multimorbidity with low BoI and BoT (segment 1) may be managing well until a new condition is added, or a condition worsens, increasing BoI and BoT. This may result in new navigational challenges (e.g., a new set of appointments and treatments). Whether the older adults with multimorbidity can cope, depends on his or her available resources. Our findings indicate that a patient grows in navigational ability as his or her conditions worsen, through interactions with the healthcare system (segment 2). However, the worsening health condition and resulting increase in appointments, treatments and medications can create new navigational challenges. A patient with severe multimorbidity may be coping well until an external event disrupts the equilibrium (moving the patient to segment 3). Conversely, a patient with navigational problems may be helped by a healthcare staff to obtain needed resources to overcome those difficulties and navigate their healthcare interactions competently (moving to segment 2). Throughout the process, a patient is constantly faced with the choice to learn or find ways to overcome navigational challenges, or to give up and disengage.

Our findings support the cumulative complexity model proposed by Shippee et al. [6]— older adults with multimorbidity are able to manage their workload (treatment burden, including attending appointments, following medication regimens and the logistics underlying these tasks) if there is good support, but there may come a time when resources are insufficient, tilting the balance into disequilibrium. For instance, although P012 had an advanced cancer and was a “full-time” patient because of his illness, he followed up on all his appointments and was very positive and invested in taking care of his health. Contrastingly, P007 had life-threatening conditions with extraordinarily strong support from his wife who assisted him in nearly all his daily activities, but they were barely managing financially and emotionally due to a recent family event. While P012 would fit in segment (2) in Fig. 2, P007 was shifting from segment 2 to 3. In essence, regardless of the severity of multimorbidity, when demands outstrip resources, older adults with multimorbidity may struggle to cope. Older adults with multimorbidity may also choose to disengage, such as P005, who did not find help within the healthcare system to enable her to achieve her healthcare priorities, or P015, who left the management of his medications and appointments completely to his wife.

### Comparison with existing literature

This study contributed to existing work on patient navigation by focusing on the needs of older adults with multimorbidity. Previous studies established that patients with multimorbidity face challenges coordinating among multiple stakeholders—hassles such as juggling multiple appointments, medication regimes and even conflicting advice from different experts [18, 30]. Our study found these challenges existed but were unevenly distributed among older adults with multimorbidity. Older adults with multimorbidity who reported the fewest hassles with navigation were retired or not working, and their medical conditions were stable enough for them to attend to their medical appointments without disruption to other activities.

We observed that those who faced chronic pain, physical mobility issues, severe burden of illness, or financial difficulties tended to encounter more navigational challenges. This may suggest that multimorbidity by itself does not necessarily impose a large navigational burden, but navigational challenges multiply when conditions are more severe, BoT increases, financial or social resources are lacking, and other patient-intrinsic factors such as illiteracy are present. Therefore, bio-psychosocial factors play a large role in helping older adults with multimorbidity navigate the healthcare system. This is in line with the findings of Boehmer et al. (2016), whose study on dialysis patients found that those with the most patient-reported disruption also experienced deficits in their physical, emotional, and financial capacity.

Our findings also support the cumulative complexity model [6]– older adults with multimorbidity who had greater BoI and BoT, along with resource scarcity, whether a lack of literacy, financial means or social support, had a greater tendency to express difficulty with navigating the healthcare system. Our findings highlighted some patient-intrinsic factors that affect navigation, such as literacy. Future work should explore other aspects of intrinsic capacity.

Foo et al.’s [20] study of management of multiple chronic diseases in Singapore identified facilitators and barriers to accessing, receiving care and self-management in the community from the perspectives of patients, caregivers and physicians. Our study extended understanding of the barriers and facilitators particularly faced by older adults with multimorbidity, such as literacy, technological literacy, age-related forgetfulness, the key role of children as caregivers, interpreters and “bridges” to the healthcare system.

Preston et al. [12] noted that patients sometimes felt compelled to ‘fit in’ to the system, whereas Vos et al. [30] noted that patients felt the need to be assertive to achieve their goals. Our study corroborates both findings. Some older adults with multimorbidity felt they had to accommodate themselves to the system, whereas others asked for help and sought to achieve their own goals through negotiation with healthcare partners.

### Implications for Research and Practice

From the findings of this study, there are several issues to be focused on and prioritised, namely redesigning the healthcare system processes, improving patient-doctor relationship involving shared decision making, language barrier and technology, risk stratification to identify patients with high risk of navigational difficulties and stoicism in Asian setting.

Firstly, it is hoped that the factors relating to the healthcare system uncovered in this study will be used to aid redesign of healthcare processes to mitigate navigational difficulties and reduce the treatment burden faced by older adults with multimorbidity. For instance, allotting doctors more time to deal with older adults with multimorbidity may be advisable, to ensure health information is well received. Clinicians may also benefit from specialised training on how to deal with older adults with multimorbidity’s specific challenges, to be able to advocate for them to receive the needed resources should the occasion arise.

Most older adults with multimorbidity also expressed a preference for a strong relationship and good rapport with their doctors, which would enable them to voice their concerns and clarify doubts. Systemic practices such as the frequent swapping around of doctors in large healthcare institutions can work against older adults with multimorbidity’s preferred strategies for navigation, which relies on familiarity and trust built up over time. When personal rapport is lacking, older adults with multimorbidity—especially the less educated—tend to withhold volunteering information to, or asking questions of, healthcare professionals. Not having a familiar doctor can therefore become a barrier to older adults with multimorbidity’s access to healthcare-related information, resulting in less-than-optimal health outcomes [31].

Older adults with multimorbidity sometimes had different priorities from their doctors. This is not unknown in the literature [18]. Healthcare professionals who took time to educate patients and discover their priorities facilitated patients’ navigation of the healthcare system by making relevant information accessible in a way they could grasp. Our findings support the drive toward patient-centric shared decision making, highlighting the need to address the issues of the most importance to the patient.

As frequent users of the healthcare system, the views of older adults with multimorbidity need to be heard more, by involving them in patient involvement advisory boards. Catering to the specific needs of older adults with multimorbidity could include designing larger fonts for medication instructions and improving communication by having language-matched healthcare professionals. For instance, increasing reliance on technology by healthcare systems presents difficulties for some older adults with multimorbidity, and healthcare providers should be mindful to offer low-tech alternatives.

Additionally, future work should consider developing frameworks for the identification of older adults with multimorbidity with higher risk of navigational difficulties. To this end, a risk stratification score or tool could be developed through further quantitative survey research using personal factors uncovered in this study. Corazza et al.’s [32] vector model for assessing patient complexity may be a useful starting point for further adaptation into a tool for clinicians to assess potentially problematic areas for patient navigation of healthcare.

Lastly, this study is set in an Asian setting in which stoicism is a strong factor among older adults who often keep their needs to themselves [33, 34]. The fear of being a burden to others can result in older adults with multimorbidity not receiving sufficient social support, as they are reluctant to discuss financial needs with children, or to bother them in any way. The language divide is an additional concern, as older adults who are not fluent in English are likely to refrain from verbalising their symptoms or worries. Moreover, a consequence of older adults with multimorbidity not wanting to “bother” their adult children is a reduction of resources available to them, potentially adding to BoT and exacerbating the challenges of healthcare navigation.

#### Strengths and limitations

The present study focused on patients with multimorbidity aged above 60 years old in an Asian cultural setting, a population that is still relatively understudied. Maximum variation sampling allowed us to uncover differences across the socioeconomic spectrum. The team approach with two coders per transcript, while time consuming, ensured that all relevant aspects of navigation had been coded, and that coding was not overly subjective.

Rigour in the analysis process was assured by having multiple coders for analyst triangulation, keeping an open attitude to confirming and disconfirming evidence, and keeping a clear audit trail [35]. Members of the research team also maintained an attitude of self-reflexivity. When the interviewer was also a clinician, particular effort was made to maintain a neutral, open attitude, bracket one’s own assumptions and initial reactions, and refrain from giving clinical advice until after the interview.

Because the study is exploratory and covers a broad area, thematic saturation rather than code saturation was sought [28]. Thematic saturation was judged to have been reached when further interviews yielded no major new themes for analysis, and a further five interviews confirmed that no new information was forthcoming. Our final sample size of 20 is in line with the findings of previous researchers, who found that 15 to 24 interviews suffice for a richly textured understanding of the topic at hand [36], although we also have in mind Braun and Clarke’s [37] warning that saturation is a concept fraught with complexity.

Finally, our recruitment strategy targeted participants who visited the polyclinics. Chronic defaulters and bed-bound patients were thereby excluded. While this was useful for our current purpose of understanding on-the-ground experiences of older adults with multimorbidity, it may have omitted a subset of patients who do not attend their appointments from being overwhelmed by their BoI and/or BoT.

## Conclusions

Older adults with multimorbidity form a population with specific characteristics and challenges, compared to younger patients with multimorbidity. They may be more likely to have difficulties with mobility and forgetfulness, and in some cases language barriers, lack of literacy or technological aversion pose challenges in their healthcare encounters. Simple tasks like appointment co-ordination and physical mobility can pose navigational difficulties for older adults with multimorbidity.

The variations among older adults with multimorbidity’s navigational experiences of the healthcare system show the need for further study of the differential needs of older adults with multimorbidity, as others have noted [38, 39]. To be truly patient-centered, healthcare providers should consider factors such as the existence of family support networks, literacy, technological literacy and the age-related challenges older adults face as they interact with the healthcare system, as well as finding ways to improve healthcare systems through personal rapport and strategies for reducing unnecessary BoT for patients with multimorbidity.

### Electronic supplementary material

Below is the link to the electronic supplementary material.

Supplementary Material 1

Supplementary Material 2

Supplementary Material 3

Supplementary Material 4

## Data Availability

All data generated or analysed during this study are included in this published article and its supplementary information files.

## Abbreviations

BoI: burden of illness
BoT: burden of treatment
DSRB: Domain Specific Review Board
MDM: minimally disruptive medicine
Pre-U: pre-university
