# Supplementary material for: How do older adults with multimorbidity navigate healthcare?: a qualitative study in Singapore
Source: BMC Prim Care. 2023 Nov 14;24:239. doi: 10.1186/s12875-023-02195-2 (PMC10644451; doi:10.1186/s12875-023-02195-2)
Supplement: Supplementary file 1 — Supplementary Material 1 [file 12875_2023_2195_MOESM1_ESM.docx]

**Additional File 1**

Interview Guide

1. Tell me about your experience living with all these different conditions

2. How do you manage the appointments of all your different conditions?

3. Can you share with us how your last visit in the polyclinic was?

4. Tell me about the best visit to the polyclinic or hospital that you have had?

5. Tell me about the worst visit to the polyclinic or hospital that you have had?

6. Can you tell us your experiences being referred from one department to another/hospital

to polyclinic/ polyclinic to A&E?

7. Do you think the staff in the clinics and hospitals who take care of you communicate the

information about your treatment and health condition to each other?

8. How do you usually feel after your appointment?

9. Have you ever encountered situations where your appointment has been changed?

10. Have you ever missed an appointment? What happened?

11. How do you find out information about the clinics or hospitals that you visit?

12. Do you have any suggestions on how the current healthcare system can be improved?
