# Supplementary material for: How do older adults with multimorbidity navigate healthcare?: a qualitative study in Singapore
Source: BMC Prim Care. 2023 Nov 14;24:239. doi: 10.1186/s12875-023-02195-2 (PMC10644451; doi:10.1186/s12875-023-02195-2)
Supplement: Supplementary file 2 — Supplementary Material 2 [file 12875_2023_2195_MOESM2_ESM.docx]

**Additional File 3**

Revised codes

Alternative medicine and GPs

Appointments

Attitude to managing own health

Caregiving responsibilities

Communication

Emotions from healthcare encounters

Facing the situation

Familiarity

Finances

Literacy

Health literacy

Medications

Others' experiences

Patient-related problems

Perception of healthcare professionals

Screening

Social support

Strategies to navigate

Suggestions

System processes

Technology - Knowledge and attitudes

Travelling to healthcare institution

Trust in healthcare professionals and healthcare system
