# Supplementary material for: How do older adults with multimorbidity navigate healthcare?: a qualitative study in Singapore
Source: BMC Prim Care. 2023 Nov 14;24:239. doi: 10.1186/s12875-023-02195-2 (PMC10644451; doi:10.1186/s12875-023-02195-2)
Supplement: Supplementary file 3 — Supplementary Material 3 [file 12875_2023_2195_MOESM3_ESM.docx]

| **Additional File 2**  Initial codes   \| Alternative medicine and GPs \| \| --- \| \| Appointments \| \| Appointment negotiation \| \| Appointment tracking \| \| Appointment-related problems \| \| Attitude to managing own health \| \| Self-management - \| \| Self-management + \| \| Effective self-management of medications \| \| Caregiving responsibilities -- to ignore \| \| Communication \| \| Availability of informational resources \| \| Healthcare system to patient communication \| \| Healthcare professional to patient communication \| \| Conflicting information or advice \| \| Effective communication \| \| Healthcare professional as educator \| \| Inter-healthcare professional communication \| \| Inter-healthcare professional -  (no communication or don't know) \| \| Inter-healthcare professional communication +  (yes communication) \| \| Language-related issues and solutions \| \| Miscommunication \| \| Negotiation \| \| Emotions from healthcare encounters \| \| Emotions- \| \| Emotions+ \| \| Facing the situation \| \| Avoidance, reluctance, denial \| \| Hopelessness, pointlessness, fatalism \| \| Humour & easy-going \| \| Normalizing, accepting attitude \| \| Familiarity \| \| Familiarity-  (unfamiliarity - physical environment) \| \| Familiarity+ \| \| Familiarity - physical environment \| \| Familiarity - routine \| \| Preference for familiar healthcare institution \| \| Finances \| \| Finances- \| \| Finances+ \| \| Literacy \| \| Health literacy- \| \| Health literacy+ \| \| Financial health literacy \| \| Knowledge about health condition \| \| Knowledge of services \| \| Understanding medications, tests & treatment \| \| Literacy- (Illiterate, poor literacy or doesn't like reading) \| \| Literacy+ \| \| Medications \| \| Medications- \| \| Medication packaging and labelling \| \| Self-adjustment of medications \| \| Medications+ (distributed) \| \| Others' experiences - distributed \| \| Patient-related problems \| \| Forgetful \| \| Illness burden \| \| Physical \| \| Psycho-emotional \| \| Not wanting to be a burden \| \| Treatment burden \| \| Perception of healthcare professionals \| \| Healthcare professional- \| \| Healthcare professional+ \| \| Personal touch \| \| Lack of time and attention given to patients \| \| Physical design and navigation \| \| Physical design- (problems navigating) \| \| Physical design+ (easy to navigate) \| \| Screening \| \| Screening- \| \| Screening+ \| \| Social support \| \| Family and friends' support- (Lacking family support) \| \| Family and friends' support+ \| \| Work environment- (non-supportive) \| \| Work environment+ (supportive) \| \| Strategies to navigate \| \| Asking for help \| \| Familiarity with navigating the system \| \| Fitting in \| \| Suggestions \| \| System processes \| \| System processes- \| \| Lack of med review \| \| Long waiting time \| \| Multiple stops- inter-institutional \| \| Multiple stops- intra-institutional \| \| No fixed doctor \| \| Over-reliance on technology \| \| Systemic inefficiencies \| \| System processes+ \| \| Coordination between different parts of healthcare system \| \| Efficiency \| \| Fixed doctor \| \| Helpful staff \| \| One stop services \| \| Short waiting time \| \| Tech - Knowledge and attitudes \| \| Tech- \| \| Tech+ \| \| Travelling to healthcare institution \| \| Convenient \| \| Inconvenient \| \| Trust in healthcare professionals and healthcare system \| \| Trust- \| \| Trust+ \| |
| --- | --- | --- | --- | --- | --- | --- | --- | --- | --- | --- | --- | --- | --- | --- | --- | --- | --- | --- | --- | --- | --- | --- | --- | --- | --- | --- | --- | --- | --- | --- | --- | --- | --- | --- | --- | --- | --- | --- | --- | --- | --- | --- | --- | --- | --- | --- | --- | --- | --- | --- | --- | --- | --- | --- | --- | --- | --- | --- | --- | --- | --- | --- | --- | --- | --- | --- | --- | --- | --- | --- | --- | --- | --- | --- | --- | --- | --- | --- | --- | --- | --- | --- | --- | --- | --- | --- | --- | --- | --- | --- | --- | --- | --- | --- | --- | --- | --- | --- | --- | --- | --- | --- | --- | --- | --- | --- | --- | --- |
|  |
