# Supplementary material for: How do older adults with multimorbidity navigate healthcare?: a qualitative study in Singapore
Source: BMC Prim Care. 2023 Nov 14;24:239. doi: 10.1186/s12875-023-02195-2 (PMC10644451; doi:10.1186/s12875-023-02195-2)
Supplement: Supplementary file 4 — Supplementary Material 4 [file 12875_2023_2195_MOESM4_ESM.docx]

**Additional File 4**

Participants’ Profile

| **Participant ID** | **Gender** | **Age** | **Education, employment, living arrangements** | **Medical conditions** |
| --- | --- | --- | --- | --- |
| P002 | Male | 77 | Primary school education, retired farmer, lives with wife and adult son | Diabetes, Eye problems, Hip Injury, Joint problems, Low haemoglobin |
| P003 | Male | 72 | Secondary school education, retired, lives with wife | Cardiovascular, Diabetes, Hypertension, Kidney disease |
| P004 | Male | 70 | Pre-university education, retired, lives with extended family | Asthma, Chronic urinary problem, Hypertension, Hyperlipidaemia, Kidney stones |
| P005 | Female | 78 | No formal education, retired food seller, lives with husband and children | Chronic musculoskeletal problems, Diabetes, Hypertension, Hyperlipidaemia, Parkinson’s disease, Stroke |
| P006 | Male | 70 | University degree, security guard, lives with wife and children | Cardiovascular disease, Glaucoma, Hyperlipidaemia, Hypertension, Hypothyroidism, Spinal cord impingement |
| P007 | Male | 62 | Secondary school education, retired security guard, lives with wife and children | Cardiovascular disease, Diabetes, Hypertension, Kidney failure (dialysis), Peripheral vascular disease, Stroke |
| P008 | Male | 77 | College degree, retired safety inspector, lives with wife and children | Age-related macular degeneration  Anxiety, Enlarged prostate, Cardiovascular disease |
| P009 | Male | 70 | No formal education, unemployed, lives with a cousin or friend (alternating domiciles) | Cardiovascular disease, Chronic musculoskeletal conditions, Chronic skin itch, Diabetes, Hypertension |
| P010 | Male | 73 | Secondary school education, retired former plumbing inspector, lives with wife and children | Asthma, Cancer, Cardiovascular disease, Cervical spondyolisis, Colon problem, Chronic urinary problem, Depression and anxiety, Diabetes, Eye problem,  Hyperlipidaemia, Hypertension, Stomach problem |
| P011 | Male | 68 | Secondary school education, bus driver, lives with wife | Hypertension, Cardiovascular disease, Diabetes, Hyperlipidaemia, Eye problem |
| P012 | Male | 68 | Secondary school education, retired, lives with wife and children | Cancer, Hypertension, Colon problem, Diabetes |
| P013 | Male | 80 | Primary school education, retired food seller, lives with wife and domestic helper | Hypertension, Chronic musculoskeletal conditions, Cardiovascular disease, Chronic urinary problem, Hyperlipidaemia, Calcium deficiency |
| P014 | Female | 69 | Primary school education, homemaker, lives with husband and children | Hypertension, Cardiovascular disease, Hyperlipidaemia |
| P015 | Male | 74 | Primary school education, currently unemployed, lives with wife and children | Hypertension, Cardiovascular disease, Diabetes, Kidney disease, Chronic urinary problem |
| P016 | Male | 82 | Pre-university education, retired teacher, lives with sister and niece | Asthma, Breathing problems, Colon problem, Diabetes, Eye problem, Hypertension, Leg pain, Shaky hands, Stomach problem |
| P017 | Female | 69 | Secondary school education, retired clerical clerk, lives with husband | Hypertension, Chronic musculoskeletal conditions, Asthma, Hyperlipidaemia |
| P018 | Female | 77 | Primary school education, Retired housekeeper, lives with children | Hypertension, Chronic musculoskeletal conditions, Arthritis, Osteoporosis, Colon problems, Diabetes, Cancer, Hyperlipidaemia, Obesity, Gout, Anaemia |
| P019 | Female | 75 | No formal education, Retired hospital staff, lives with children | Hypertension, Chronic musculoskeletal conditions, Cardiovascular disease, Stomach problems, Diabetes, Kidney disease, Chronic urinary problem |
| P020 | Female | 85 | Primary school education, Retired factory worker, lives with children | Hypertension, Chronic musculoskeletal conditions, Arthritis, Osteoporosis, Stroke, Hyperlipidaemia |
| P021 | Female | 80 | No formal education, Retired factory worker, lives with children | Hypertension, Arthritis, Diabetes, Hyperlipidaemia, Eye problem, Schizophrenia |
